# Comparison of Immunotherapy, Chemotherapy, and Chemoimmunotherapy in Advanced Pulmonary Lymphoepithelioma-Like Carcinoma： A Retrospective Study

**DOI:** 10.3389/fonc.2022.820302

**Published:** 2022-02-14

**Authors:** Yi Xiao, Jinyuan He, Shaoning Luo, Min Dong, Wei Li, Gaijiao Liu, Hongjie Chen, Xiongwen Yang, Shaohong Huang

**Affiliations:** ^1^ Department of Thoracocardiac Surgery, The Third Affiliated Hospital of Sun Yat-sen University, Guangzhou, China; ^2^ Department of Emergency Medicine, The Third Affiliated Hospital of Sun Yat-sen University, Guangzhou, China; ^3^ Department of Oncology, The Third Affiliated Hospital of Sun Yat-sen University, Guangzhou, China; ^4^ Department of Urology, Affiliated Hospital of Xizang Minzu University, Xianyang, China; ^5^ Department of Anesthesiology, The First Affiliated Hospital of Sun Yat-sen University, Guangzhou, China; ^6^ Department of Traditional Chinese Medicine, The Third Affiliated Hospital of Sun Yet-sen University, Guangzhou, China; ^7^ Department of Surgical Oncology, The First People’s Hospital of ChenZhou City, Chenzhou, China; ^8^ College of Medicine, South China University of Technology, Guangzhou, China

**Keywords:** lymphoepithelioma-like carcinoma, chemotherapy, immunotherapy, chemoimmunotherapy, prognosis free survival

## Abstract

Pulmonary lymphoepithelioma-like carcinoma (pLELC) is a rare subtype of lung cancer that is associated with the Epstein-Barr virus in Asia. Due to the lack of prospective studies, the best first-line treatment and survival outcomes remain unclear. Herein, This study investigated the efficacy and safety of different treatment regimens for advanced pLELC. This retrospective study included 68 patients with advanced pLELC from two centers in China. Patients were divided into three groups according to different first-line treatments: chemotherapy (n=49, 72.1%), immunotherapy (n=7, 10.3%), and chemoimmunotherapy (n=12,17.6%). The primary endpoint of this study was the 2-year progression-free survival (PFS) of each group. The results show that the median PFS was 6.9 months (range, 2.3–not estimable) in the chemotherapy group, 11.0 months (range, 2–not estimable) in the immunotherapy group, and 11.8 months (range, 6–not estimable) in the chemoimmunotherapy group. There was a significant difference in 2-year PFS between the chemoimmunotherapy group and the chemotherapy group (hazard ratio, 0.38, 95% confidence interval: 0.18-0.78, log-rank *P=*0.007). The most frequent grade 3-4 adverse event in the chemotherapy and chemoimmunotherapy groups was myelosuppression (10/49 [22.4%] and 4/12 [33.3%], respectively). The most frequent grade 3-4 adverse events in the immunotherapy group were diarrhea (1/7, 14.8%) and hepatotoxicity (1/7, 14.8%). Chemoimmunotherapy had the highest 2-year PFS as a first-line treatment for advanced pLELC compared to chemotherapy and immunotherapy. This study suggests that chemoimmunotherapy may be the best first-line treatment for patients with advanced pLELC.

## Introduction

Pulmonary lymphoepithelioma-like carcinoma (pLELC) is a rare pathological subtype of lung cancer (1), which has been shown to be closely associated with Epstein-Barr virus (EBV) infection (2). pLELC has unique clinicopathological features, such as predominance in younger non-smokers in Asia and heavy lymphocytic infiltration (1, 3, 4).

Lung tumor cells frequently express high levels of programmed death ligand 1 (PD-L1), which is the ligand of the programmed death-1 (PD-1) receptor on T cells, allowing tumors to directly inhibit the host immune response by inhibiting the proliferation and function of T cells (5–9). Moreover, the treatment landscape of lung cancer has dramatically changed due to immunotherapy. One of the most notable is the PD-L1/PD-1 checkpoint inhibitor (10). Several studies have confirmed that PD-L1 expression is higher in patients with pLELC than in those with other types of lung cancer (11–13), which may mean that immunotherapy may benefit patients with advanced pLELC.

Nasopharyngeal carcinoma (NPC) is also associated with EBV infection and has a high propensity for regional and distant metastases, while it is very sensitive to radiation and chemotherapy (14). A common feature of advanced EBV-positive NPC is the dense infiltration of lymphocytes in the tumor stroma and positive PD-L1 expression in tumor cells, making it an attractive target for immunotherapy (14). Immunotherapy has shown promise in patients with advanced nasopharyngeal cancer (15, 16).

Currently, there is no standard treatment for pLELC. Most patients diagnosed with pLELC are usually present at an early stage and undergo complete resection (17). However, in advanced cases, multimodal treatment including systemic chemotherapy and radiation is often required (18). Furthermore, immunotherapy has shown great advantages as a first-line treatment of advanced lung cancer (19–24), but the effect of immunotherapy in advanced pLELC has rarely been reported (25–28). In this study, we included patients with advanced pLELC who received first-line immunotherapy and reviewed our initial experience with the use of this regimen in patients with advanced pLELC. To our knowledge, this is the first study on first-line immunotherapy in patients with pLELC.

## Methods

### Patients

**Figure 1:**
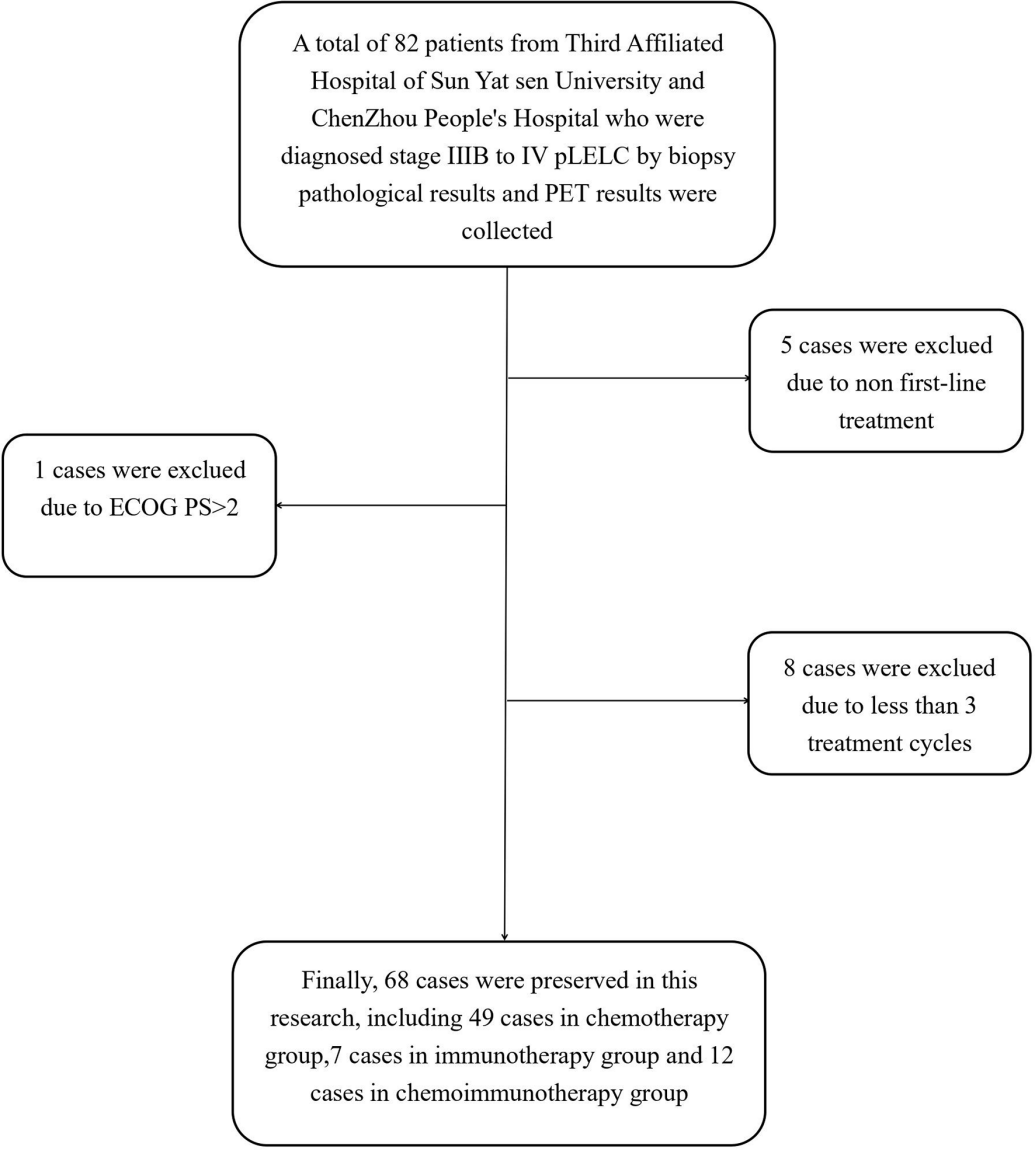
Flow-chart of this study.

In this study, patients with stage IIIB to IV pLELC who were diagnosed *via* biopsy and computed tomography/positron emission tomography at The Third Affiliated Hospital of Sun Yat-sen University and The People’s Hospital of Chenzhou City between January 2012 and August 2020 were retrospectively investigated. The inclusion criteria were as follows: confirmed pathological diagnosis of pLELC, Eastern Cooperative Oncology Group performance status (ECOG PS) ≤ 2, and ≥2 regular treatment cycles. The exclusion criteria were as follows: had previous treatment after diagnosis, non first-line treatment, or lacked completed radiology or follow-up data. Finally, 68 patients were included in the study (**Figure 1**).

The collected clinical data of the patients included sex, age, smoking history, ECOG PS, Epstein-Barr encoding region (EBER), PD-L1 expression (22C3 PD-L1 antibody, Dako, Denmark), tumor location, clinical TNM (cTNM) stage, treatment mode, treatment cycle, treatment-related adverse events (TRAEs), and PFS. cTNM was determined according to the 8^th^ edition of the lung cancer staging system of the American Joint Committee on Cancer (29). Based on the different treatment modes collected, the patients were divided into chemotherapy, immunotherapy, and chemoimmunotherapy groups.

### Treatment Regimen

The chemotherapy group received platinum-based chemotherapy in combination with other non-platinum anticancer agents as doublet chemotherapy. Subsequently, non-platinum anti-cancer agents were administered as maintenance in some patients. In the first four treatment cycles, the chemoimmunotherapy group received a triple-drug regimen, including platinum, non-platinum anti-cancer agents, and PD-1 monoclonal antibody. Subsequently, the PD-1 monoclonal antibody was given as maintenance. All patients in the immunotherapy group received PD-1 monoclonal antibody monotherapy only. All treatment cycles were continuous and 3–4 weeks apart.

### Treatment Evaluation

Patients underwent a CT scan every 6–8 weeks to evaluate the treatment effect. The change in tumor burden was assessed using the Response Evaluation Criteria in Solid Tumors (RECIST, version 1.1) (30). All radiology data were assessed by two doctors to obtain the most precise results. Considering the possibility of tumor pseudoprogression after patients received immunotherapy, disease progression determined in the immunotherapy and chemoimmunotherapy groups required proof by two consecutive radiology examinations.

TRAEs were assessed using the National Cancer Institute Common Terminology Criteria for Adverse Events (version 5.0) (31).

### Statistical Analysis

All statistical analyses in this study were performed using R software v3.61 (https://www.r-project.org/). Considering that the earliest date of immunotherapy patient data that could be collected was July, 2017, the primary endpoint was determined as the 2-year PFS in this study. PFS was defined as the time from the initiation of treatment to definite tumor progression or death. All follow-up data were collected until September 1, 2021. Patient age was transformed into a categorical variable (≥65 years old or <65 years old). Fisher’s exact test was used to determine significant differences between groups for categorical variables. PFS curves were completed using the Kaplan–Meier method and assessed using the log-rank test. The median and 95% confidence intervals (CIs) and p-values from log-rank tests are reported in figures. All P values were two-sided, and values <0.05 were considered statistically significant.

## Results

### Patient Characteristics

**Figure 2:**
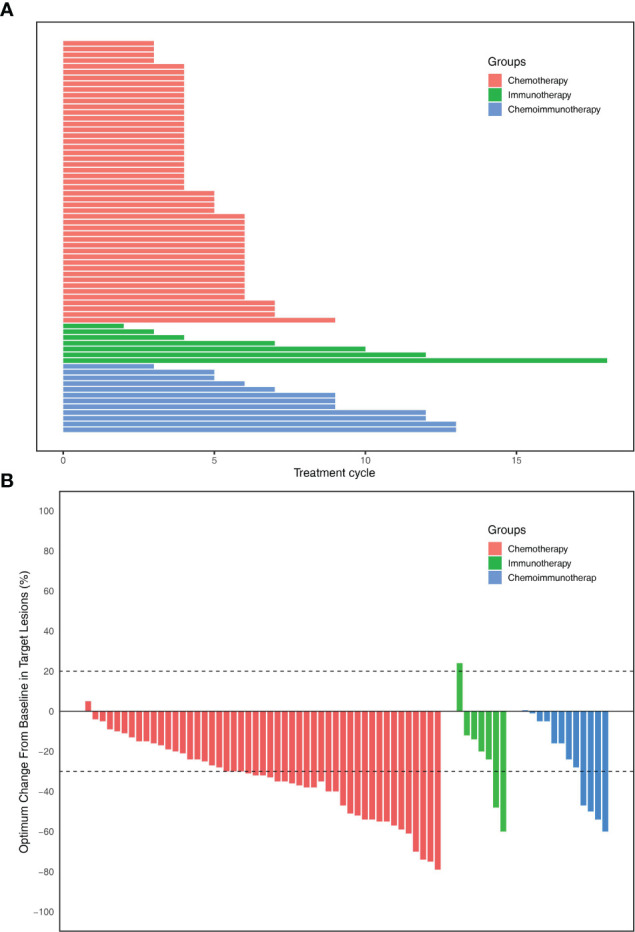
**(A)** Total number of treatment cycles of each patients. **(B)** The optimum change in individual patients of three groups.

A total of 68 patients were included in this study (**Table 1**). The median follow-up time was 7.9 months. Of the 68 patients, 49 (72.1%) underwent chemotherapy, 7 (10.3%) underwent immunotherapy, and 12 (17.6%) underwent chemoimmunotherapy (**Table 1**). There were 45 (91.8%), 5 (71.4%), and 11 (91.7%) patients with more than three treatment cycles in the chemotherapy, immunotherapy, and chemoimmunotherapy groups, respectively (**Figure 2A**). Of the 49 patients in the chemotherapy group, 24 (49.0%) received gemcitabine plus platinum, 12 (24.5%) received paclitaxel plus platinum, and 13 (26.5%) received pemetrexed plus platinum **(Appendix 1)**. In the immunotherapy group, 5 (71.4%) patients received pembrolizumab and 2 (28.6%) received sintilimab **(Appendix 1)**. 8 (66.7%), 2 (16.7%), and 2 (16.7%) patients in the chemoimmunotherapy group received pembrolizumab plus gemcitabine + platinum, pembrolizumab plus pemetrexed + platinum, and pembrolizumab plus paclitaxel + platinum, respectively (**Appendix 1**). In the group of patients who received pembrolizumab plus gemcitabine + platinum, 3 patients reached PR and 5 reached SD (**Appendix 4**). In the group of patients that received pembrolizumab plus pemetrexed + platinum and pembrolizumab plus paclitaxel + platinum, 1 reached PR and 3 reached SD. There were 8 (11.8%) stage IIIB/IIIC patients and 60 (88.2%) stage IV patients. Of the 60 stage IV patients, 12 (20%), 19 (31.7%), 20 (33.3%), 29 (48.3%), and 5 (8.3%) patients had lung, liver, pleural, bone, and adrenal gland metastases **(Appendix 1)**, respectively.

**Table 1:** General clinicopathological characteristics of patients with different treatment methods.

Characteristics	Entire cohort (N=68)			p
	Chemotherapy (N=49)	Immunotherapy (N=7)	Chemoimmunotherapy (N=12)	
Gender, No. (%)				0.926
Female	27 (55.1)	4 (57.1)	6 (50.0)	
Male	22 (44.9)	3 (42.9)	6 (50.0)	
Age, No. (%)				0.472
>65	6 (12.2)	1 (14.3)	3 (25.0)	
≤65	43 (87.8)	6 (85.7)	9 (75.0)	
Smoking history, No. (%)				0.122
Current/former	10 (20.4)	4 (57.1)	2 (16.7)	
Never	39 (70.6)	3 (42.9)	10 (83.3)	
EBER, No. (%)				
+	11 (22.4)	1 (14.3)	5 (41.7)	0.710
++	13 (25.6)	2 (28.6)	2 (17.6)	
+++	25 (51.0)	4 (57.1)	5 (41.7)	
ECOG PS, No. (%)				1
0	3 (6.1)	0 (0)	1 (8.3)	
1	46 (93.9)	7 (100.0)	11 (91.7)	
Tumor location, No. (%)				0.923
Upper right lung	6 (12.2)	0 (0)	2 (16.7)	
Right middle lung	9 (18.4)	2 (28.5)	2 (16.7)	
Lower right lung	10 (20.4)	3 (42.9)	2 (16.7)	
Upper left lung	10 (20.4)	1 (14.3)	3 (25.0)	
Lower left lung	14 (28.6)	1 (14.3)	3 (25.0)	
T, No. (%)				
T1/T2	12 (24.5)	2 (28.6)	3 (25.0)	1
T3/T4	37 (75.5)	5 (71.4)	9 (75.0)	
N, No. (%)				
N0/N1	5 (10.2)	0 (0)	0 (0)	0.333
N2	22 (44.4)	1 (14.7)	5 (41.7)	
N3	22 (44.4)	6 (85.3)	7 (58.3)	
Tumor stage, No. (%)				0.577
Stage IIIB/IIIC	5 (10.2)	1 (14.7)	2 (16.7)	
Stage IV	44 (89.8)	6 (85.3)	10 (83.3)	

There were no significant differences in sex, age, smoking history, EBER expression, ECOG PS, tumor location, cT, cN, or tumor stage among the three groups (**Table 1**).

### Treatment Efficacy

**Figure 3:**
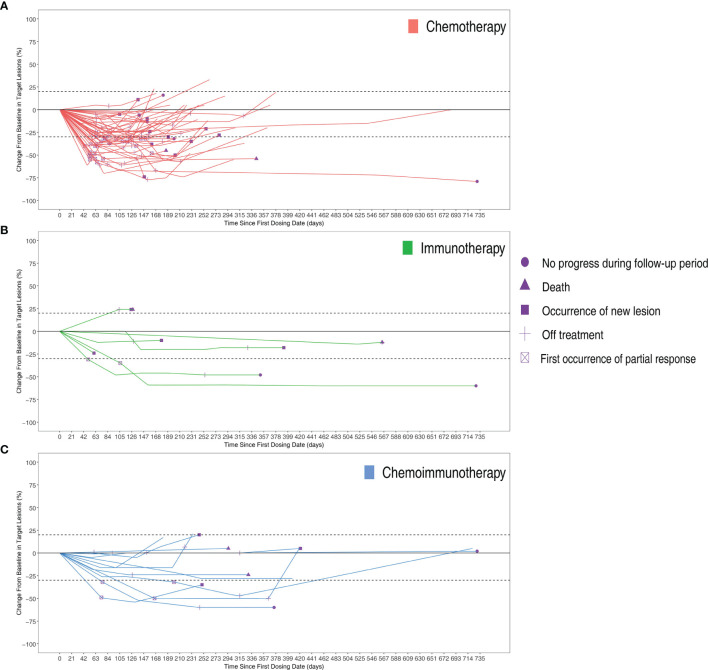
Changes from baseline in target lesions of per patients in three groups over time with **(A)** Chemotherapy group **(B)** Immunotherapy group **(C)** Chemoimmunotherapy group.

**Figure 4:**
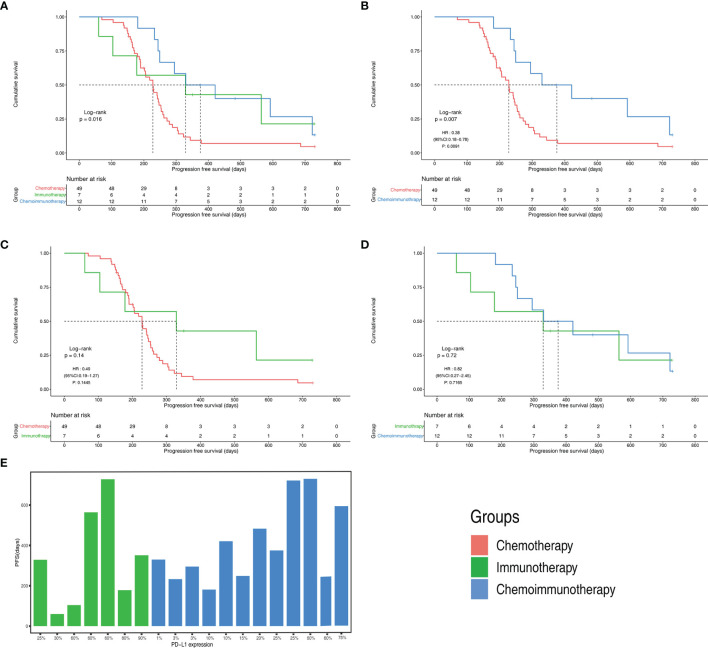
Progression free survival(PFS) of different groups **(A)** PFS Kaplan-Meier curves of chemotherapy, immunotherapy and chemoimmunotherapy groups **(B)** PFS Kaplan-Meier curves of chemotherapy group and chemoimmunotherapy groups **(C)** PFS Kaplan-Meier curves of chemotherapy group and immunotherapy groups **(D)** PFS Kaplan-Meier curves of immunotherapy and chemoimmunotherapy groups **(E)** PFS with different PD-L1 expression in immunotherapy and chemoimmunotherapy groups. CI, confidence interval; HR, hazard ratio.

The objective response rates were 61.2% in the chemotherapy group, 28.6% in the immunotherapy group, and 33.3% in the immunochemotherapy group (**Figure 2B**). The disease control rates were 100%, 85.7%, and 100% in the chemotherapy, immunotherapy, and chemoimmunotherapy groups, respectively. No patient reached complete remission (CR) in any group. One patient in each of the three groups had no disease progression until the end of follow-up (**Figure 3**). New lesions developed in 12 (24.5%), 1 (14.3%), and 2 (16.7%) patients in the chemotherapy, immunotherapy, and chemoimmunotherapy groups, respectively (**Figure 3**). The median PFS (mPFS) was 6.9 months (range, 2.3 months to not estimable) in the chemotherapy group, 11.0 months (range, 2 months to not estimable) with immunotherapy, and 11.8 months (range, 6.0 months to not estimable) with immunochemotherapy (**Figure 4**). The results of the log-rank comparison revealed that compared to the chemotherapy group, the chemoimmunotherapy group was significantly associated with a better 2-year PFS (**Figure 4B**, hazard ratio, 0.38, 95% confidence interval: 0.18-0.78, log-rank *P=*0.007). However, there was no significant difference in 2-year PFS between the immunotherapy and chemotherapy groups (**Figure 4C**, P = 0.14) or between the immunotherapy and chemoimmunotherapy groups (**Figure 4D**, P = 0.72).

### PD-L1 Expression With PFS

All patients in both the immunotherapy and chemoimmunotherapy groups were positive for PD-L1 (**Figure 4E**). The mPFS of patients in the immunotherapy group with PD-L1 expression greater than or equal to 50% was 11.7 months and that of patients with PD-L1 expression lower than 50% was 6.5 months. In immunotherapy group and chemoimmunotherapy group, patients with PD-L1 expression greater than or equal to 50% had an mPFS of 19.7 months and those with PD-L1 expression lower than 50% had an mPFS of 11.0 months.

### PD-L1 Expression With Treatment Response

There were 3 (75%) patients in the chemoimmunotherapy group and 5 (71.4%) in the immunotherapy group with PD-L1 expression ≥ 50% (**Appendix 3**). The ECOG PS of all patients in the immunotherapy group was 1. In the subgroup of the immunotherapy group with PD-L1 expression ≥ 50%, 3 (60%) patients were women, 4 (80%) were ≤ 65 years of age, 2 (40%) reached PR, 2 (40%) reached SD, and 1 (20%) reached PD. In the subgroup of the immunotherapy group with PD-L1 expression < 50%, there was 1 male patient and 1female patient; all the patients were ≤ 65 years old and reached SD. In the subgroup of the chemoimmunotherapy group with PD-L1 expression ≥ 50%, 2 (66.7%) were men, all patients were ≤ 65 years old; the ECOG PS were 1, 1 patient reached PR, and 2 had the optimal response of SD. In the subgroup of the chemoimmunotherapy group with PD-L1 expression < 50%, 5 (55.6%) were men, 3 (33.3%) were > 65 years old, 1 (11.1%) had ECOG PS of 0, 8 (88.9%) had ECOG PS of 1, 3 (33.3%) reached PR, and 6 (66.7%) reached SD.

### Safety

There were no instances of grade 5 TRAEs in this study. Grade 3/4 TRAEs were reported in 14 (28.6%), 2 (28.6%), and 4 (33.3%) patients in the chemotherapy, immunotherapy, and chemoimmunotherapy groups, respectively (**Table 2**). Myelosuppression was the most common grade 3/4 TRAE in the chemotherapy group (20.4%, 10/49) and the chemoimmunotherapy group (33.3%, 4/12).

**Table 2:** Treatment-related grade 3/4 adverse events of three groups.

Side effect	Chemotherapy Grade 3/4 No. (%)	Immunotherapy Grade 3/4 No. (%)	Chemoimmunotherapy Grade 3/4 No. (%)
Diarrhea	0 (0)	1 (14.3)	0 (0)
Fever	2 (4)	0 (0)	0 (0)
Rash	1 (2)	0 (0)	0 (0)
Alopecia	3 (6.1)	0 (0)	0 (0)
Hemoglobin decreased	4 (8.1)	0 (0)	1 (8.3)
Leukoreduction	5 (10.2)	0 (0)	2 (16.6)
Thrombocytopenia	1 (2.0)	0 (0)	1 (8.3)
Hepatotoxicity	0 (0)	1 (14.3)	0 (0)
Hemoptysis	0 (0)	0 (0)	1 (8.3)

## Discussion

This is the first study to evaluate the efficacy and safety of first-line immunotherapy with or without chemotherapy in advanced pLELC. Our results showed that the 2-year PFS with chemoimmunotherapy was significantly better than that of chemotherapy (hazard ratio, 0.38, 95% confidence interval: 0.18-0.78, log-rank *P=*0.007), with an mPFS that was 5 months longer than chemotherapy.

Most patients in this cohort were young, non-smoking females, which is a unique feature of pLELC compared to other histological types of lung cancer. Consistent with previous studies, pLELC originating in the right upper lung was rare (25, 32). In locally advanced pLELC, the effect of chemoradiotherapy is better than chemotherapy alone (18, 25). However, most patients in our cohort had metastases and were not candidates for radiotherapy. Previous studies have indicated that compared with other NSCLCs, the mutations of EGFR, ALK, and MET were extremely low in patients with pLELC, suggesting that familiar typical driver mutations may not play a critical role in pLELC (33, 34). Furthermore, PD-L1 expression is higher in patients with pLELC than in those with other types of lung cancer (11–13). Currently, there is no optimal treatment for pLELC owing to the lack of clinical trials. The selection of first-line treatment options is primarily empirical and is usually based on the histological classification (e.g., non-squamous or non-specific) of non-small cell lung cancer (NSCLC). Prospective clinical trials considering pLELC would be impractical because of the rarity of this tumor type. Meanwhile, first-line immunotherapy has shown promise in advanced lung cancer and NPC. Therefore, we believe that first-line immunotherapy with or without chemotherapy is worth trying in advanced pLELC.

In the KEYNOTE-024 study, the mPFS was 10.3 months (95% CI, 6.7 to not reach) in the pembrolizumab group and 6.0 months (95% CI, 4.2-6.2) in the chemotherapy group. PFS was significantly longer in the pembrolizumab group than in the chemotherapy group (HR=0.50; 95% CI, 0.37-0.68; P<0.001) (19, 20). In the KEYNOTE-189 study, the mPFS was 9.0 (8.1-9.9) months and 4.9 (4.7-5.5) months in the pembrolizumab plus chemotherapy and chemotherapy groups, respectively (HR=0.48; 95% CI, 0.40-0.58), with estimated 2-year PFS rates of 20.5% and 1.5%, respectively. Furthermore, a PFS benefit with the addition of pembrolizumab was observed regardless of PD-L1 expression (21, 22). These two prospective trials demonstrated a significant PFS benefit of pembrolizumab with or without chemotherapy in patients with advanced NSCLC. For EBV-associated cancer, previous studies have shown that the treatment schemes of pLELC and NPC are similar (18, 35). Similarly, in advanced NPC, nivolumab and pembrolizumab have shown promising antitumor activity and manageable safety (15, 16).

The pattern of response observed in this study is consistent with the response to PD-1 inhibitors reported in other cancers (36). In the path graph, two patients showed delayed responses after the first 4 months of treatment. Only one non-responder progressed within the first 2 months of treatment, and none of the patients showed a dramatic increase in tumor size. In addition, there were no patients with CR in our cohort, possibly due to large tumor size at baseline or the small sample size.

More than 95% of adults worldwide are infected with EBV, but there are regional differences in the prevalence of EBV-associated cancers (37, 38). Epidemiological studies have shown that EBV infection is associated with LELC in Asian populations, but not in Western populations (39). The common drivers of lung cancer, such as *EGFR* mutations and *ALK* rearrangement, are rarely detected in pLELC, indicating that the key carcinogens of LELC are not tobacco exposure or somatic cell driver mutations (13). Furthermore, the mutation spectrum of pLELC was found to be distinct from those of other subtypes of NSCLC and EBV^+^ NPC, with unique frequently mutated genes and widespread copy number variation loss (13). All patients in our cohort were EBER-positive. In addition, several previous studies of pLELC have demonstrated a potential association between circulating EBV DNA and survival (18, 40, 41). However, only three patients in our cohort had baseline EBV data. We cannot speculate on the relationship between baseline EBV levels and response to drug therapy or survival during immunotherapy. Future studies require larger sample sizes and more comprehensive virological studies to address the relationship between EBV infection and the efficacy of checkpoint inhibitors in pLELC.

This study has several limitations. First, the fact that this is a retrospective study with small sample sizes is an obvious weakness, even though we included data from two centers. Second, immunotherapy is a relatively new therapeutic approach, and the first patient in our cohort to receive first-line immunotherapy began in 2017. Follow-up may be insufficient, and there was no mature overall survival in this study. Third, immunotherapy drugs in our cohort included nivolumab, pembrolizumab, and sintilimab. Due to the small sample size, we were unable to compare the efficacy differences between different drugs. In this study, the limited sample size makes it difficult to interpret inferences regarding the correlation between PD-L1 expression and treatment response or survival. Finally, we only recorded grade 3-4 adverse events in detail; mild adverse events were not presented due to incomplete records. Therefore, we do not know if there are specific adverse events in pLELC. In conclusion, we evaluated a small number of patients and demonstrated that immune checkpoint inhibitors may be a promising first-line treatment option for advanced pLELC. Large-scale clinical trials are required to determine the best treatment for the disease.

## Data Availability Statement

The raw data supporting the conclusions of this article will be made available by the authors, without undue reservation.

## Ethics Statement

This study was approved by the ethics committee of the Third Affiliated Hospital of Sun Yat sen University and The First People’s Hospital of ChenZhou city. All patients were informed and consented.

## Author Contributions

Conception and design: YX, XWY, SHH, and MD. Administrative support: SHH and HJC. Provision of study materials or patients: XWY, YX, SNL, JH, WL, and GJL. Collection and assembly of data: YX and WL. Data analysis and interpretation: YX and XWY. Manuscript writing: All authors. All authors contributed to the article and approved the submitted version.

## Funding

This study was supported by Guangdong Natural Science Foundation (No. 2021A1515012548).

## Conflict of Interest

The authors declare that the research was conducted in the absence of any commercial or financial relationships that could be construed as a potential conflict of interest.

## Publisher’s Note

All claims expressed in this article are solely those of the authors and do not necessarily represent those of their affiliated organizations, or those of the publisher, the editors and the reviewers. Any product that may be evaluated in this article, or claim that may be made by its manufacturer, is not guaranteed or endorsed by the publisher.

## References

[1] Travis WD Brambilla E Nicholson AG Yatabe Y Austin JHM Beasley MB. The 2015 World Health Organization Classification of Lung Tumors: Impact of Genetic, Clinical and Radiologic Advances Since the 2004 Classification. J Thorac Oncol (2015) 10(9):1243–60. doi: 10.1097/JTO.0000000000000630 26291008

[2] Begin LR Eskandari J Joncas J Panasci L. Epstein-Barr Virus Related Lymphoepithelioma-Like Carcinoma of Lung. J Surg Oncol (1987) 36(4):280–3. doi: 10.1002/jso.2930360413 2826922

[3] Ngan RK Yip TT Cheng WW Chan JK Cho WC Ma VW. Circulating Epstein-Barr Virus DNA in Serum of Patients With Lymphoepithelioma-Like Carcinoma of the Lung: A Potential Surrogate Marker for Monitoring Disease. Clin Cancer Res (2002) 8(4):986–94. doi: 10.1093/carcin/23.4.669 11948104

[4] Gomez-Roman JJ Martinez MN Fernandez SL Val-Bernal JF. Epstein-Barr Virus-Associated Adenocarcinomas and Squamous-Cell Lung Carcinomas. Mod Pathol (2009) 22(4):530–7. doi: 10.1038/modpathol.2009.7 19252476

[5] Akbay EA Koyama S Carretero J Altabef A Tchaicha JH Christensen CL. Activation of the PD-1 Pathway Contributes to Immune Escape in EGFR-Driven Lung Tumors. Cancer Discov (2013) 3(12):1355–63. doi: 10.1158/2159-8290.CD-13-0310 PMC3864135 24078774

[6] Dong H Strome SE Salomao DR Tamura H Hirano F Flies DB. Tumor-Associated B7-H1 Promotes T-Cell Apoptosis: A Potential Mechanism of Immune Evasion. Nat Med (2002) 8(8):793–800. doi: 10.1038/nm730 12091876

[7] Ribas A. Adaptive Immune Resistance: How Cancer Protects From Immune Attack. Cancer Discov (2015) 5(9):915–9. doi: 10.1158/2159-8290.CD-15-0563 PMC4560619 26272491

[8] Tumeh PC Harview CL Yearley JH Shintaku IP Taylor EJ Robert L. PD-1 Blockade Induces Responses by Inhibiting Adaptive Immune Resistance. Nature (2014) 515(7528):568–71. doi: 10.1038/nature13954 PMC4246418 25428505

[9] Suresh S Chen B Zhu J Golden RJ Lu C Evers BM. eIF5B Drives Integrated Stress Response-Dependent Translation of PD-L1 in Lung Cancer. Nat Cancer (2020) 1(5):533–45. doi: 10.1038/s43018-020-0056-0 PMC7511089 32984844

[10] Carlisle JW Steuer CE Owonikoko TK Saba NF. An Update on the Immune Landscape in Lung and Head and Neck Cancers. CA Cancer J Clin (2020) 70(6):505–17. doi: 10.3322/caac.21630 32841388

[11] Wu Q Wang W Zhou P Fu Y Zhang Y Shao YW. Primary Pulmonary Lymphoepithelioma-Like Carcinoma Is Characterized by High PD-L1 Expression, But Low Tumor Mutation Burden. Pathol Res Pract (2020) 216(8):153043. doi: 10.1016/j.prp.2020.153043 32703503

[12] Xie Z Liu L Lin X Xie X Gu Y Liu M. A Multicenter Analysis of Genomic Profiles and PD-L1 Expression of Primary Lymphoepithelioma-Like Carcinoma of the Lung. Mod Pathol (2020) 33(4):626–38. doi: 10.1038/s41379-019-0391-9 PMC7113185 31659278

[13] Chen B Zhang Y Dai S Zhou P Luo W Wang Z. Molecular Characteristics of Primary Pulmonary Lymphoepithelioma-Like Carcinoma Based on Integrated Genomic Analyses. Signal Transduct Target Ther (2021) 6(1):6. doi: 10.1038/s41392-020-00382-6 33414372 PMC7791019

[14] Chen YP Chan ATC Le QT Blanchard P Sun Y Ma J. Nasopharyngeal Carcinoma. Lancet (2019) 394(10192):64–80. doi: 10.1016/S0140-6736(19)30956-0 31178151

[15] Hsu C Lee SH Ejadi S Even C Cohen RB Le Tourneau C. Safety and Antitumor Activity of Pembrolizumab in Patients With Programmed Death-Ligand 1-Positive Nasopharyngeal Carcinoma: Results of the KEYNOTE-028 Study. J Clin Oncol (2017) 35(36):4050–6. doi: 10.1200/JCO.2017.73.3675 28837405

[16] Ma BBY Lim WT Goh BC Hui EP Lo KW Pettinger A. Antitumor Activity of Nivolumab in Recurrent and Metastatic Nasopharyngeal Carcinoma: An International, Multicenter Study of the Mayo Clinic Phase 2 Consortium (NCI-9742). J Clin Oncol (2018) 36(14):1412–8. doi: 10.1200/JCO.2017.77.0388 PMC5941615 29584545

[17] Sathirareuangchai S Hirata K. Pulmonary Lymphoepithelioma-Like Carcinoma. Arch Pathol Lab Med (2019) 143(8):1027–30. doi: 10.5858/arpa.2018-0149-RS 30672338

[18] Lin Z Fu S Zhou Y Zhang X Chen C He LN. First-Line Platinum-Based Chemotherapy and Survival Outcomes in Locally Advanced or Metastatic Pulmonary Lymphoepithelioma-Like Carcinoma. Lung Cancer (2019) 137:100–7. doi: 10.1016/j.lungcan.2019.09.007 31568886

[19] Reck M Rodriguez-Abreu D Robinson AG Hui R Csoszi T Fulop A. Pembrolizumab Versus Chemotherapy for PD-L1-Positive Non-Small-Cell Lung Cancer. N Engl J Med (2016) 375(19):1823–33. doi: 10.1056/NEJMoa1606774 27718847

[20] Reck M Rodriguez-Abreu D Robinson AG Hui R Csoszi T Fulop A. Updated Analysis of KEYNOTE-024: Pembrolizumab Versus Platinum-Based Chemotherapy for Advanced Non-Small-Cell Lung Cancer With PD-L1 Tumor Proportion Score of 50% or Greater. J Clin Oncol (2019) 37(7):537–46. doi: 10.1200/JCO.18.00149 30620668

[21] Gadgeel S Rodriguez-Abreu D Speranza G Esteban E Felip E Domine M. Updated Analysis From KEYNOTE-189: Pembrolizumab or Placebo Plus Pemetrexed and Platinum for Previously Untreated Metastatic Nonsquamous Non-Small-Cell Lung Cancer. J Clin Oncol (2020) 38(14):1505–17. doi: 10.1200/JCO.19.03136 32150489

[22] Gandhi L Rodriguez-Abreu D Gadgeel S Esteban E Felip E De Angelis F. Pembrolizumab Plus Chemotherapy in Metastatic Non-Small-Cell Lung Cancer. N Engl J Med (2018) 378(22):2078–92. doi: 10.1056/NEJMoa1801005 29658856

[23] Hellmann MD Paz-Ares L Bernabe Caro R Zurawski B Kim SW Carcereny Costa E. Nivolumab Plus Ipilimumab in Advanced Non-Small-Cell Lung Cancer. N Engl J Med (2019) 381(21):2020–31. doi: 10.1056/NEJMoa1910231 31562796

[24] Paz-Ares L Ciuleanu TE Cobo M Schenker M Zurawski B Menezes J. First-Line Nivolumab Plus Ipilimumab Combined With Two Cycles of Chemotherapy in Patients With Non-Small-Cell Lung Cancer (CheckMate 9LA): An International, Randomised, Open-Label, Phase 3 Trial. Lancet Oncol (2021) 22(2):198–211. doi: 10.1016/S1470-2045(20)30641-0 33476593

[25] Zhou N Lin Y Peng X Wang Y Wang Y. Thorough Survey and Analysis of Pulmonary Lymphoepithelioma-Like Carcinoma in Macau and Multimodality Treatment for Advanced Disease. Lung Cancer (2019) 138:116–23. doi: 10.1016/j.lungcan.2019.10.004 31683094

[26] Narayanan A Knollmann FD Walby JAS Lim S Gandara DR Riess JW. EBV-Positive Primary Pulmonary Lymphoepithelioma-Like Carcinoma Response to PD-L1 Blockade. Clin Lung Cancer (2019) 20(3):e238–41. doi: 10.1016/j.cllc.2018.12.015 30679078

[27] Qiu ZX Zhou P Wang K. Primary Pulmonary Lymphoepithelioma-Like Carcinoma Response Favorably To Nivolumab: A Case Report. Onco Targets Ther (2019) 12:8595–600. doi: 10.2147/OTT.S219512 PMC6802557 31802895

[28] Wu Z Xian X Wang K Cheng D Li W Chen B. Immune Checkpoint Blockade Therapy May Be a Feasible Option for Primary Pulmonary Lymphoepithelioma-Like Carcinoma. Front Oncol (2021) 11:626566. doi: 10.3389/fonc.2021.626566 33981599 PMC8110193

[29] Goldstraw P Chansky K Crowley J Rami-Porta R Asamura H Eberhardt WE. The IASLC Lung Cancer Staging Project: Proposals for Revision of the TNM Stage Groupings in the Forthcoming (Eighth) Edition of the TNM Classification for Lung Cancer. J Thorac Oncol (2016) 11(1):39–51. doi: 10.1016/j.jtho.2015.09.009 26762738

[30] Eisenhauer EA Therasse P Bogaerts J Schwartz LH Sargent D Ford R. New Response Evaluation Criteria in Solid Tumours: Revised RECIST Guideline (Version 1.1). Eur J Cancer (2009) 45(2):228–47. doi: 10.1016/j.ejca.2008.10.026 19097774

[31] Common Terminology Criteria for Adverse Events (CTCAE) V5.0 (2021). Available at: https://ctep.cancer.gov/protocoldevelopment/electronic_applications/docs/CTCAE_v5_Quick_Reference_8.5x11.pdf.

[32] Yu X Wen Y Qin R Lin Y Zhang X Wang W. Prognosis and Distribution of Lymph Nodes Metastases in Resectable Primary Pulmonary Lymphoepithelioma-Like Carcinoma: A Large Cohort From a Single Center. Thorac Cancer (2018) 9(3):360–7. doi: 10.1111/1759-7714.12586 PMC5832476 29327422

[33] Yin K Feng HB Li LL Chen Y Xie Z Lv ZY. Low Frequency of Mutation of Epidermal Growth Factor Receptor (EGFR) and Arrangement of Anaplastic Lymphoma Kinase (ALK) in Primary Pulmonary Lymphoepithelioma-Like Carcinoma. Thorac Cancer (2020) 11(2):346–52. doi: 10.1111/1759-7714.13271 PMC6997003 31794146

[34] Hong S Liu D Luo S Fang W Zhan J Fu S. The Genomic Landscape of Epstein-Barr Virus-Associated Pulmonary Lymphoepithelioma-Like Carcinoma. Nat Commun (2019) 10(1):3108. doi: 10.1038/s41467-019-10902-w 31311932 PMC6635366

[35] Qin Y Gao G Xie X Zhu Z Guan W Lin X. Clinical Features and Prognosis of Pulmonary Lymphoepithelioma-Like Carcinoma: Summary of Eighty-Five Cases. Clin Lung Cancer (2019) 20(3):e329–37. doi: 10.1016/j.cllc.2018.12.014 30683631

[36] Hoos A. Development of Immuno-Oncology Drugs - From CTLA4 to PD1 to the Next Generations. Nat Rev Drug Discov (2016) 15(4):235–47. doi: 10.1038/nrd.2015.35 26965203

[37] Kanda T Yajima M Ikuta K. Epstein-Barr Virus Strain Variation and Cancer. Cancer Sci (2019) 110(4):1132–9. doi: 10.1111/cas.13954 PMC6447851 30697862

[38] de Martel C Georges D Bray F Ferlay J Clifford GM. Global Burden of Cancer Attributable to Infections in 2018: A Worldwide Incidence Analysis. Lancet Glob Health (2020) 8(2):e180–90. doi: 10.1016/S2214-109X(19)30488-7 31862245

[39] Castro CY Ostrowski ML Barrios R Green LK Popper HH Powell S. Relationship Between Epstein-Barr Virus and Lymphoepithelioma-Like Carcinoma of the Lung: A Clinicopathologic Study of 6 Cases and Review of the Literature. Hum Pathol (2001) 32(8):863–72. doi: 10.1053/hupa.2001.26457 11521232

[40] Xie M Wu X Wang F Zhang J Ben X Zhang J. Clinical Significance of Plasma Epstein-Barr Virus DNA in Pulmonary Lymphoepithelioma-Like Carcinoma (LELC) Patients. J Thorac Oncol (2018) 13(2):218–27. doi: 10.1016/j.jtho.2017.10.031 29191777

[41] Ngan RK Yip TT Cheng WW Chan JK Cho WC Ma VW. Clinical Role of Circulating Epstein-Barr Virus DNA as a Tumor Marker in Lymphoepithelioma-Like Carcinoma of the Lung. Ann NY Acad Sci (2004) 1022:263–70. doi: 10.1196/annals.1318.041 15251971

