# Supplementary material for: Comparison of Immunotherapy, Chemotherapy, and Chemoimmunotherapy in Advanced Pulmonary Lymphoepithelioma-Like Carcinoma： A Retrospective Study
Source: Front Oncol. 2022 Feb 14;12:820302. doi: 10.3389/fonc.2022.820302 (PMC8882604; doi:10.3389/fonc.2022.820302)
Supplement: Supplementary file 1 [file Table_1.docx]

| Groups | Therapeutic drugs No.(%) | | |
| --- | --- | --- | --- |
| Chemotherapy  (N=49) | Gemcitabine + platinum | paclitaxel + platinum | pemetrexed + platinum |
|  | 24 (49.0%) | 12 (24.5%) | 13 ( 26.5%) |
| Immunotherapy  (N=7) | pembrolizumab | sintilimab |  |
|  | 5 (71.4%) | 2 (28.6%) |  |
| Chemoimmunotherapy  (N=12) | pembrolizumab + gemcitabine + platinum | Pembrolizumab + pemetrexed + platinum | pembrolizumab + paclitaxel + platinum |
|  | 8 (66.7%) | 2 (16.7%) | 2 (16.7%) |

Appendix 1: Details of treatment regimen in each groups
