# Supplementary material for: Comparison of Immunotherapy, Chemotherapy, and Chemoimmunotherapy in Advanced Pulmonary Lymphoepithelioma-Like Carcinoma： A Retrospective Study
Source: Front Oncol. 2022 Feb 14;12:820302. doi: 10.3389/fonc.2022.820302 (PMC8882604; doi:10.3389/fonc.2022.820302)
Supplement: Supplementary file 2 [file Table_2.docx]

|  | Chemotherapy | Immunotherapy | Chemoimmunotherapy |
| --- | --- | --- | --- |
| Metastatic sites |  |  |  |
| Lung/Pleura | 24 | 3 | 4 |
| Liver | 14 | 3 | 2 |
| Bone | 20 | 4 | 5 |
| Adrenal gland | 4 | 0 | 1 |

Appendix 2: Metastatic site of patients on baseline
