# Supplementary material for: Comparison of Immunotherapy, Chemotherapy, and Chemoimmunotherapy in Advanced Pulmonary Lymphoepithelioma-Like Carcinoma： A Retrospective Study
Source: Front Oncol. 2022 Feb 14;12:820302. doi: 10.3389/fonc.2022.820302 (PMC8882604; doi:10.3389/fonc.2022.820302)
Supplement: Supplementary file 3 [file Table_3.doc]

**Appendix 3:** General clinicopathological characteristics and treatment response of different PD-L1 expression level subgroup in immunotherapy group and chemoimmunotherapy group

| Characteristics | Immunotherapy  (N=7) | | Chemoimmunotherapy  (N=12) | |
| --- | --- | --- | --- | --- |
|  | PD-L1 expression  (＜50%)  （N=2） | PD-L1 expression  (≥50%)  （N=5） | PD-L1 expression  (＜50%)  （N=9） | PD-L1 expression  (≥50%)  （N=3） |
| Gender, No. (%) |  |  |  |  |
| Female | 1 (50) | 3 (60) | 4 (44.4) | 1 (33.3) |
| Male | 1 (50) | 2 (40) | 5 (55.6) | 2 (66.7) |
| Age, No. (%) |  |  |  |  |
| ＞65 | 0 | 1 (20) | 3 (33) | 0 |
| ≤65 | 2 (100) | 4 (80) | 6 (67) | 3 (100) |
| Tumor stage, No. (%) |  |  |  |  |
| Stage IIIB/IIIC | 1 (50) | 0 | 1 (11.1) | 1 (33.3) |
| Stage IV | 1 (50) | 5 (100) | 8 (88.9) | 2 (66.6) |
| EBER, No. (%) |  |  |  |  |
| + | 0 | 1 (20) | 6 (66.7) | 1 (33.3) |
| ++ | 0 | 2 (40) | 0 | 1 (33.3) |
| +++ | 2 (100) | 2 (40) | 3 (33.3) | 1 (33.3) |
| ECOG PS, No. (%) |  |  |  |  |
| 0 | 0 | 0 | 1 (11) | 0 |
| 1 | 2 (100) | 5 (100) | 8 (89) | 3 (100) |
| Optimal effect, No. (%) |  |  |  |  |
| CR | 0 | 0 | 0 | 0 |
| PR | 0 | 2 (40) | 3 (33) | 1 (33) |
| SD | 2 (100) | 2 (40) | 6 (67) | 2 (67) |
| PD | 0 | 1 (20) | 0 | 0 |

EBER: Epstein-Barr encoding region; ECOG PS: Eastern Cooperative Oncology Group performance status; CR: complete response; PR: partial response; SD: stable disease; PD: progressive disease
