# Supplementary material for: Comparison of Immunotherapy, Chemotherapy, and Chemoimmunotherapy in Advanced Pulmonary Lymphoepithelioma-Like Carcinoma： A Retrospective Study
Source: Front Oncol. 2022 Feb 14;12:820302. doi: 10.3389/fonc.2022.820302 (PMC8882604; doi:10.3389/fonc.2022.820302)
Supplement: Supplementary file 4 [file Table_4.doc]

**Appendix 4**: Optimal response of GC/immunotherapy group and Non-GC/immunotherapy group

| Treatment response | GC/immunotherapy group  N=8 | Non-GC/immunotherapy group  N=4 |
| --- | --- | --- |
|
| CR, No. (%) | 0 | 0 |
| PR, No. (%) | 3 (37.5) | 1 (25) |
| SD, No. (%) | 5 (62.5) | 3 (75) |
| PD, No. (%) | 0 | 0 |

In Non-GC/immunotherapy group, 2 patients received chemoimmunotherapy with pemetrexed/platinum, 2 patients received chemoimmunotherapy with paclitaxel/platinum.

GC: gemcitabine plus platinum
